# The Microbial Diversity of Cabbage Pest *Delia radicum* Across Multiple Life Stages

**DOI:** 10.3389/fmicb.2020.00315

**Published:** 2020-02-27

**Authors:** Tijs J. M. van den Bosch, Cornelia U. Welte

**Affiliations:** Department of Microbiology, Institute for Water and Wetland Research, Radboud University, Nijmegen, Netherlands

**Keywords:** 16S rRNA sequencing, microbial diversity, cabbage root fly, *Delia radicum*, community profiling, DADA2, phyloseq

## Abstract

The cabbage root fly *Delia radicum* is a worldwide pest that causes yield losses of many common cabbage crops. The bacteria associated with *D. radicum* are suggested to influence the pest status of their host. In this study, we characterized insect-associated bacteria of *D. radicum* across multiple life stages and of their diet plant (turnip, *Brassica rapa* subsp. *rapa*) by sequencing the V3–V4 region of 16S rRNA genes using the Illumina MiSeq platform. In total, over 1.2M paired-end reads were obtained, identifying 1006 bacterial amplicon sequence variants (ASVs) in samples obtained from the eggs, larvae, pupae and adults of *D. radicum*, as well as turnips that were either fresh or infested with *D. radicum* larvae. The microbial community in *D. radicum* was dominated by *Wolbachia*, a common endosymbiont of arthropods which we found in all of the investigated insect samples, with the pupal stage having the highest relative abundance. Moderate amounts of Firmicutes were found only in adult *D. radicum* flies, but not in previous life stages. Actinobacteria were mostly found on the eggs and on the skin of fresh plants on which the eggs were deposited. These plants also harbored a large amount of *Pseudomonas*. The bacterial diversity of the healthy turnip was low, whereas the microbial community of decaying turnips that were heavily infested by *D. radicum* larvae and showing symptoms of advanced soft rot was characterized by a high bacterial diversity. Taken together, this work provides insights into the bacterial communities associated with the cabbage pest *D. radicum* and its associated disease symptoms.

## Introduction

Studies that characterize the microbial community profiles of different insect species are gaining increasing attention, yet not much is known about the microbiome of the cabbage root fly *Delia radicum.* This crop pest can cause devastating yield losses on many popular vegetable crops, e.g., cabbage, kohlrabi or rapeseed (Soroka et al., 2004; Dosdall et al., 2012). The females of this species deposit their eggs onto or near the stem of cruciferous plants and emerging larvae bore into root tissue. The larvae generally stay inside the plant tissue, only to move into the soil just before pupation and to subsequently emerge as adults (Smith, 1927). Metamorphosis causes drastic changes in the anatomy and physiology of the insects, and studies on other insects are reporting that it can also have drastic effects on the associated microbiome (Geib et al., 2009; Hammer et al., 2014; Hammer and Moran, 2019).

The importance of symbiotic bacteria has long been well established in a multitude of Eukaryotic models. Also in insects, symbionts have been shown to have important functions in host physiology, e.g., by supplying essential amino acids, conferring resistance against insecticides, or even preventing predation by other insects (Buchner, 1965; Oliver et al., 2005; Kikuchi et al., 2012). The majority of the associations between insect guts and bacteria are of a facultative nature and appear to perform context-dependent functions (Mason et al., 2018). Gut microbes are thus suggested to mediate insect interactions with plant defensive compounds, effectively improving herbivore performance in real time, and between generations (Mason et al., 2018). Diet is an important factor in determining the fitness of any insect, and is a factor that can easily be manipulated in laboratory cultures. A near-aseptic laboratory culture of the diamondback moth (*Plutella xylostella*) showed increased fitness after being treated with antibiotics and then inoculated with symbiotic bacteria that were resistant to that antibiotic (Somerville et al., 2019). Although this study showed that certain gut symbionts of *P. xylostella* can have significant impact on fitness, the intestinal tracts of caterpillars are typically characterized by short transit times and a high pH which make microbial colonization difficult, and wild Lepidoptera (caterpillars) have been suggested to typically lack resident gut symbionts (Hammer et al., 2017).

Regarding the microbiome of *D. radicum*, a comprehensive analysis of the gut-associated microbial community associated to different developmental life stages compared to the healthy and invaded feed plant is lacking. A 2006 study by Lukwinski and co-workers (Lukwinski et al., 2006) was the first to analyze the gut microbial community of eggs, larval midgut and feces using a culturing approach. They found that the culturable midgut microbial community was primarily composed of Gammaproteobacteria. The microbial communities of eggs and fecal material were only analyzed regarding their colony forming units, and no further identification was performed. Subsequently, Welte et al. (2016a) performed a metagenome analysis of the larval gut microbial community which provided a more detailed view, largely congruent with the findings by Lukwinski et al. (2006), showing Gammaproteobacteria as the most abundant group of gut-associated microorganisms and several others (*Wolbachia, Bacteroidetes, Firmicutes, Actinobacteria*, Betaproteobacteria) in lower abundance. Bili et al. (2016) analyzed the microbial communities associated to adult flies of two different populations of *D. radicum*. They found that both *D. radicum* populations showed *Wolbachia* as most dominant associated microbial group. Both populations also harbored a low amount of Firmicutes (≤1% abundance), and one of the two populations’ microbiomes showed an abundant fraction of *Gluconacetobacter* (18% abundance).

In this study, our objective was to compare the microbial communities associated to the different life stages of *D. radicum* as well as healthy and invaded food sources. We profiled the microbial community of the eggs, larvae, pupae and adults. We also identified the major differences of bacterial communities that were associated with healthy the feed plants *Brassica rapa* subsp. *rapa* pre-predation, compared to plants that were macerated after being invaded by *D. radicum* larvae. This rot only developed in turnips that were used as egg laying substrate by *D. radicum*, suggesting that it is an effect caused either directly by the larvae or by larva-associated bacteria, which is a mechanism that is well described in *Drosophila* (Blum et al., 2013). Bacterial isolates from *D. radicum* have already shown the potential for metabolizing secondary metabolites that are associated with the diet plant of *D. radicum* (Welte et al., 2016a). Our results lead to the description of the microbial communities of multiple life stages of *D. radicum* as well as that of the environment that is created in the diet plant of the larvae. This work provides novel data for the research of symbioses in largely unstudied holometabolous insect groups, and may ultimately even aid in identifying transmission routes of insect and plant-associated bacteria as well as potential novel targets in microbial pest control.

## Materials and Methods

### *Delia radicum* Rearing

The eggs that formed the starting colony of *D. radicum* were obtained from Wageningen University (Laboratory of Entomology, Prof. Joop van Loon). Flies were kept in an entomology cage (60 × 60 × 120 cm) with *ad libitum* access to water and a 1:1:1 mixture of dry food consisting of yeast extract, skim milk powder and sucrose. Cages were kept in a laboratory without humidity control at room temperature and under natural lighting. For breeding purposes, a small plastic container (12 × 12 × 6 cm, now referred to as egg box) was filled with 1 cm of wetted river sand upon which a turnip (*B. rapa* subsp. *rapa*) was placed to facilitate egg deposition. Egg boxes were moved out of the entomology cage after a week and the larvae were left to feed on the supplied turnip for approximately 4 weeks until pupation. Pupae were then separated from the river sand by flooding the egg box and sieving the water, after which the pupae were placed back into an entomology cage until eclosion, marking the beginning of a new generation.

### Sample Acquisition

#### Turnip (TURN1-5)

Skin of turnips (*B. rapa* subsp. *rapa*) was cut superficially with a surface-sterilized razor blade, approximately 2 mm thick. Approximately 5 g of this material was homogenized in liquid nitrogen by the use of mortar and pestle. Approximately 0.25 g of homogenous material was used downstream with PowerSoil DNA extraction.

#### Decaying Turnip Tissue (PULP1-5)

Approximately 200 mg samples of macerated tissue from a *D. radicum* maggot-infested turnip were transferred directly to a PowerSoil tube with a surface-sterile spatula.

#### Eggs (EGGS1-5)

Approximately 200 *D. radicum* eggs were collected by flooding the egg boxes with (non-sterilized) demiwater, and collecting the runoff in a thin-necked volumetric flask in order to concentrate the floating eggs at the top of the flask. They were then collected with a Whatman filter, and placed in a filter holder. Another Whatman filter was placed on top and the eggs were washed with 5 mL of the following solutions using a syringe: milliQ water, 6.5% bleach, 70% ethanol, and milliQ water. Subsequently, the Whatman filters containing the eggs were homogenized by freezing in liquid nitrogen and then crushing by mortar and pestle. The homogenate was used downstream in DNA extraction using the PowerSoil kit.

#### Larvae (MAGG1-10)

Twenty larvae were collected with sterile forceps and stored at −20°C until DNA extraction. Prior to DNA extraction, all were washed in 5 mL sterile MQ water, then in 5 mL 70% ethanol, then in 5.5–7.5% active chlorine bleach, and lastly rinsed in 5 mL MQ again. Subsequently, all whole larvae were homogenized by mortar and pestle in liquid nitrogen.

#### Pupae (PUPA1-5)

40-100 pupae were collected by flooding an egg box with tap water, and running the top liquid containing the pupae through a generic sieve. Prior to DNA extraction, all pupae were surface-sterilized in an identical fashion to the larval samples. Subsequently, all whole pupae were homogenized by mortar and pestle in liquid nitrogen.

#### Flies

30-40 flies were collected from entomology cages with an aspirator and subsequently killed with chloroform. Flies were sexed based on the morphology of the abdomen; flies with swollen abdomens that are characteristic for gravid females (FLYF1-5) were designated as females and flies with shriveled abdomens (FLYM1-5) were designated as males. Individuals with inconclusive exteriors were discarded. Flies were surface-sterilized in an identical fashion to the larval samples and subsequently homogenized by mortar and pestle in liquid nitrogen. For detailed descriptions on corresponding origins and amounts of biological material that was used per sample, as well as the DNA concentrations after extraction, we refer to the metadata table (Supplementary Table 1).

### Sequencing and Analysis of the 16S rRNA Gene V3–V4 Region

After sample preparation, all 40 samples were weighed and DNA was extracted using the DNeasy PowerSoil kit (Qiagen) according to the manufacturer’s protocol. Due to low DNA concentrations, the samples were concentrated using a SpeedVac Vacuum concentrator. Samples were submitted to BaseClear (Leiden, the Netherlands) for paired-end sequencing of the V3–V4 region on the Illumina MiSeq system, where the primers CCTACGGGNGGCWGCAG and GACTACHVGGGTATCTAATCC were used for the generation of the V3–V4 region amplicon (Klindworth et al., 2013). 80 paired-end FASTQ read sequence files (two per sample) were generated using bcl2fastq2 version 2.18 and initial quality assessment was based on data passing the Illumina Chastity filtering. Reads containing PhiX control signal were removed using an in-house filtering protocol by BaseClear. Second quality assessment was based on the remaining reads using the FASTQC quality control tool version 0.11.5. Raw reads were delivered demultiplexed and without non-biological nucleotides (i.e., sequencing primers, adapters, linkers), and the primers used for amplification of the V3–V4 region were manually removed by deleting the first 17 NTs from forward reads and the first 21 NTs from reverse reads using simple unix commands prior to preprocessing. Preprocessing of the sequencing data was done using the DADA2 pipeline (Callahan et al., 2016). Taxonomic assignment of the reads was done up to the species level with DADA2 using the Silva non-redundant database version 128 (Yilmaz et al., 2014). A phylogenetic tree of the 1006 ASVs that were resolved by the DADA2 pipeline was created by RAxML (Stamatakis, 2006). Data visualization and analysis were performed using the phyloseq package (McMurdie and Holmes, 2013). Chao1 was used for a measure of estimated richness, whereas the Shannon-Weaver index provides more information about community composition and evenness by considering relative abundances (Kim et al., 2017). In order to compare sample groups and test the null hypothesis that the dispersion of the groups as defined by measure space are equivalent for all groups, we performed permutational multivariate analysis of variance (PERMANOVA) using adonis from the ‘vegan’ package in R (Oksanen et al., 2019). Distance matrices were built with the Bray-Curtis method of vegdist and multilevel pairwise comparisons were performed in 999 permuations with a wrapper for adonis (Martinez Arbizu, 2019).

## Results

### Preprocessing

Paired-end sequencing of the V3–V4 region of the 16S rRNA gene resulted in a total of 1,249,018 reads from 40 samples. Quality score profiles were typical for MiSeq sequencing, with high quality scores for >250 cycles in the forward direction and ∼200 cycles in the reverse direction (Supplementary Figure 1). Rarefaction curves indicated that the coverage of the bacterial diversity was sufficient and we therefore chose not to subsample to an equal depth per sample. Recently developed methods allowed us to resolve amplicon sequence variants (ASVs) instead of resorting to the more classical construction of molecular operational taxonomic units (OTUs). The benefits of this method include higher resolution and reproducibility, and allows for simple merging between independently processed datasets (Callahan et al., 2017). Unless stated otherwise, the data and analyzes of this manuscript excludes reads that were taxonomically assigned to mitochondria, chloroplast, or where the taxonomic assignment was not resolved at the phylum level.

### Bacterial Community Composition

**FIGURE 1:**
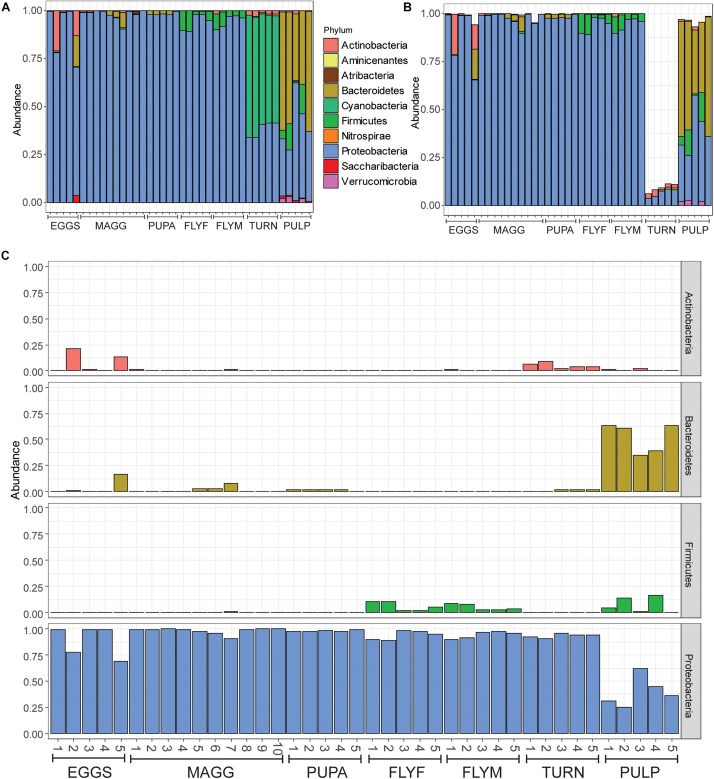
**(A)** All reads represented as relative abundance, after filtering of low-abundance reads (mean(x) > 5e–5). **(B)** Bar plot after removal of reads where Class was designated as “Chloroplast” or “Unidentified,” or where Family was designated as “Mitochondria.” Note that over 80% of reads were removed from samples in the “turnip” sample group, which can be explained by the high number of chloroplasts within the tissue. **(C)** Relative reads of Actinobacteria, Bacteroidetes, Firmicutes, and Proteobacteria in all samples, faceted by phylum. EGGS, *Delia radicum* eggs; MAGG, *Delia radicum* larvae; PUPA, *D. radicum* pupae; FLYF, Gravid female adults of *D. radicum***;** FLYM, Male adults of *D. radicum***;** TURN, Skin of fresh, non-infested *Brassica rapa* subsp. *rapa*; PULP, Macerated tissue of a *D. radicum*-infested individual of *B. rapa* subsp. *rapa*.

**FIGURE 2:**
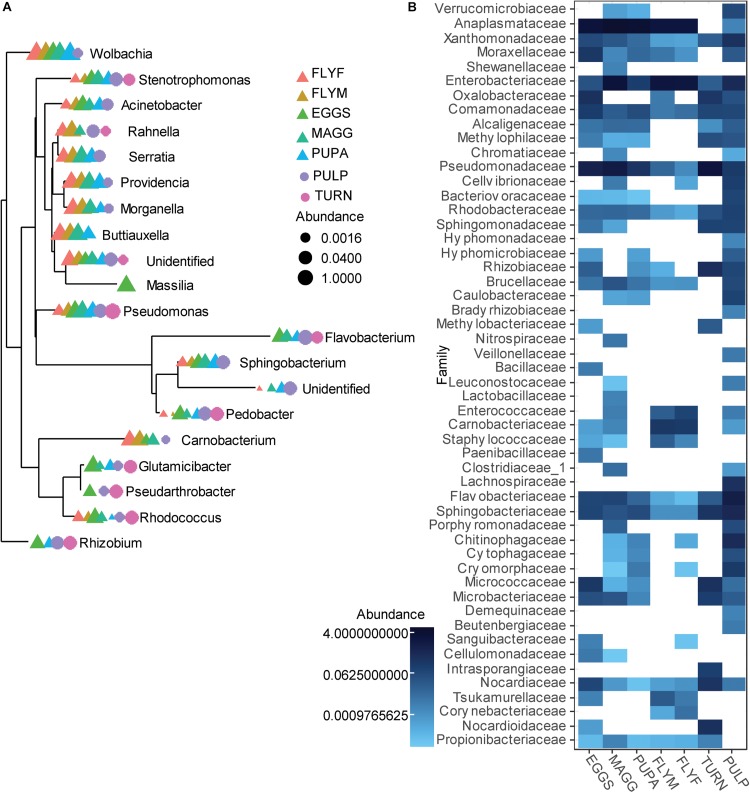
**(A)** Overview of the 20 most abundant genera associated with a laboratory culture of *Delia radicum* including samples from multiple life stages, and from fresh and decayed turnips. Data was first agglomerated at the genus level, then merged per sample. **(B)** Heatmap that shows the relative abundance of reads per sample groups, where abundances were merged at the Family level. EGGS, *D. radicum* eggs; MAGG, *D. radicum* larvae; PUPA, *D. radicum* pupae; FLYF, Gravid female adults of *D. radicum*; FLYM, Male adults of *D. radicum*; TURN, Skin of fresh, non-infested *Brassica rapa* subsp. *rapa*; PULP, Macerated tissue of a *D. radicum*-infested individual of *B. rapa* subsp. *rapa*.

A total of 1006 amplicon sequence variants (ASVs) were identified in the entire dataset. Filtering ASVs with a relative abundance mean smaller than 5e–5 left a total of 357 abundant ASVs across ten phyla (Figure 1A). All *D. radicum* samples, regardless of life stage, were largely dominated by Proteobacteria. Most of the sequence data gathered from the samples of intact turnips (∼90%) consisted of ASVs that came from chloroplasts and were thus not suitable for the determination of its microbiome (Figure 1B). Moderate amounts of Firmicutes appeared in adult *D. radicum* flies, but not in previous life stages (Figure 1C). The remaining turnip ASVs were mostly classified as Proteobacteria and Actinobacteria. Samples of decaying turnip tissue contained high numbers of Bacteroidetes as well as Proteobacteria (Figure 1C), and uniquely harbored Verrucomicrobia ASVs (not shown). Contamination from chloroplast was not apparent in these samples. An overview of the 20 most abundant genera across all samples is presented in Figure 2A, and a heatmap depicting the relative abundances of different bacterial families across sample groups can be seen in Figure 2B.

### Gammaproteobacteria

**FIGURE 3:**
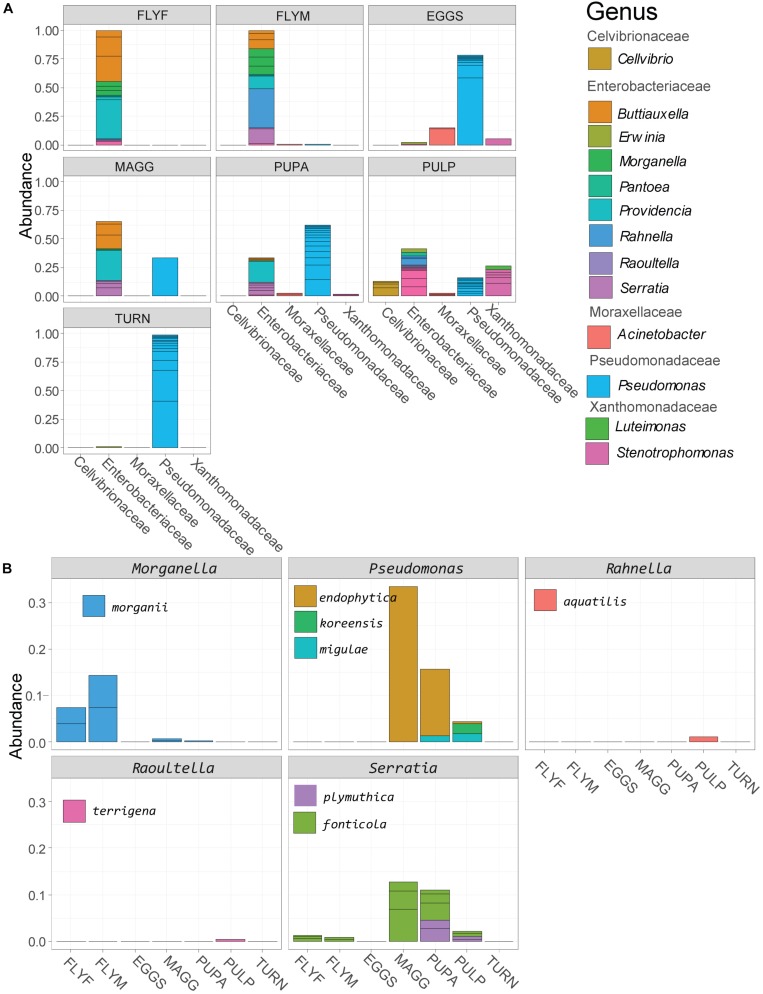
**(A)** Relative abundance of reads in the class of Gammaproteobacteria, agglomerated by sample group. **(B)** Gammaproteobacterial strains that could be identified to the species level based on sequencing of the V3–V4 region. MAGG, *Delia radicum* larvae; PUPA, *D. radicum* pupae; FLYF, Gravid female adults of *D. radicum*; FLYM, Male adults of *D. radicum*; TURN, Skin of fresh, non-infested *Brassica rapa* subsp. *rapa*; PULP, Macerated tissue of a *D. radicum*-infested individual of *B. rapa* subsp. *rapa*.

Gammaproteobacterial reads made up 31% of the reads in the dataset and we decided to use this subset for a deeper analysis (Figure 3A). We found that almost all adult fly ASVs in the Gammaproteobacteria class were affiliated with the family Enterobacteriaceae, whereas larvae and pupae also contained considerable amounts (30-62%) of *Pseudomonas* reads. High relative abundances (>75%) of ASVs in the *Pseudomonas* genus were found in *D. radicum* egg samples and in fresh turnip skin samples. The Gammaproteobacterial ASVs found in the decayed turnip tissue represented all five families. One of the advantages of generating amplicon sequence variants with the DADA2 pipeline rather than 97%-identity-based OTU clustering is that this higher-resolution method allows for the taxonomic assignment of species-level and sometimes even strain-level variants. Strain-level variants can be a source of functional diversity and can represent specialists adapted to particular hosts or environments, the identification of which would otherwise be obscured by OTU clustering (Kwong et al., 2017). The taxonomy of 34 ASVs could be determined to the species level, of which 22 were Gammaproteobacteria, and 8 of these were categorized as abundant (relative abundance > 5e-4) (Figure 3B). *Morganella morganii* was found primarily in the adult flies and in small amounts in larvae and pupae. *Morganella* sp. were also found among the 20 most abundant genera (Figure 2A), so it can be concluded that different species of this genus were present in all samples, among which *Morganella morganii*. The genus *Rahnella* was also detected among the 20 most abundant genera (Figure 2A) found in all life stages of *D. radicum* apart from eggs. One ASV identified down to the species level was *Rahnella aquatilis* that was primarily found in the decayed turnip tissue, but was previously also reported inside the gut of certain species of longicorn beetles in Korea (Park et al., 2007). Three *Pseudomonas* species (*P. endophytica, P. migulae, P. koreensis*) and two species of *Serratia (S. fonticola* and *S. plymuthica)* could be identified in larvae, pupae and decayed turnip tissue. Representatives of both genera were also found to be very abundant in all life stages of *D. radicum* (Figure 2A) but the methods employed in this study did not allow for deeper taxonomic identification. Although sequencing of the V3–V4 region did not allow strain-level resolution, it is interesting to note that the strain *Serratia plymuthica* 3Rp8 was previously isolated from the rhizosphere of *Brassica napus* L. (Adam et al., 2016).

### Wolbachia

**FIGURE 4:**
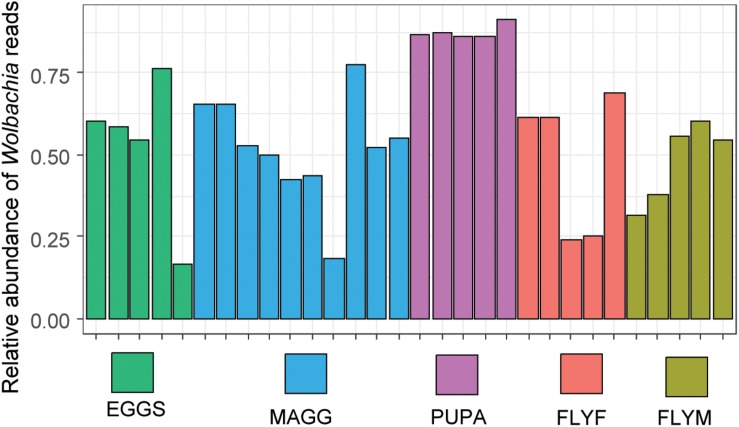
Relative abundance of reads assigned to the genus of *Wolbachia* in *Delia radicum* samples. MAGG, *D. radicum* larvae; PUPA, *D. radicum* pupae; FLYF, Gravid female adults of *D. radicum*; FLYM, Male adults of *D. radicum*; TURN, Skin of fresh, non-infested *Brassica rapa* subsp. *rapa*; PULP, Macerated tissue of a *D. radicum*-infested individual of *B. rapa* subsp. *rapa*.

The genus *Wolbachia* comprises endosymbiotic bacteria and is known to infect a wide variety of arthropods and nematodes (Serbus et al., 2008). In our dataset, ASVs that were taxonomically assigned to the genus *Wolbachia* made up 52% of the total reads, which includes ten non-insect samples, nine of which were devoid of *Wolbachia* reads. Since we extracted the DNA of multiple individuals for every *D. radicum* sample, the infection rates of the culture cannot be determined with this data and it is possible that not 100% of individuals carry *Wolbachia*. One out of five samples of decaying turnip (“PULP4”) yielded 34 reads of *Wolbachia*, which is most likely due to the accidental inclusion of larval tissue during sampling. A total of nine different ASVs were designated as *Wolbachia*, one being represented by 478723 reads, whereas the other eight contained only between 2 and 25 reads. In all likelihood, the detection of the latter variants was the result of sequencing errors and/or artifacts in the ASV-calling algorithm of DADA2. Between-sample variation of relative abundance of *Wolbachia* reads in *D. radicum* life stages is presented in Figure 4.

### Sample Diversity

**FIGURE 5:**
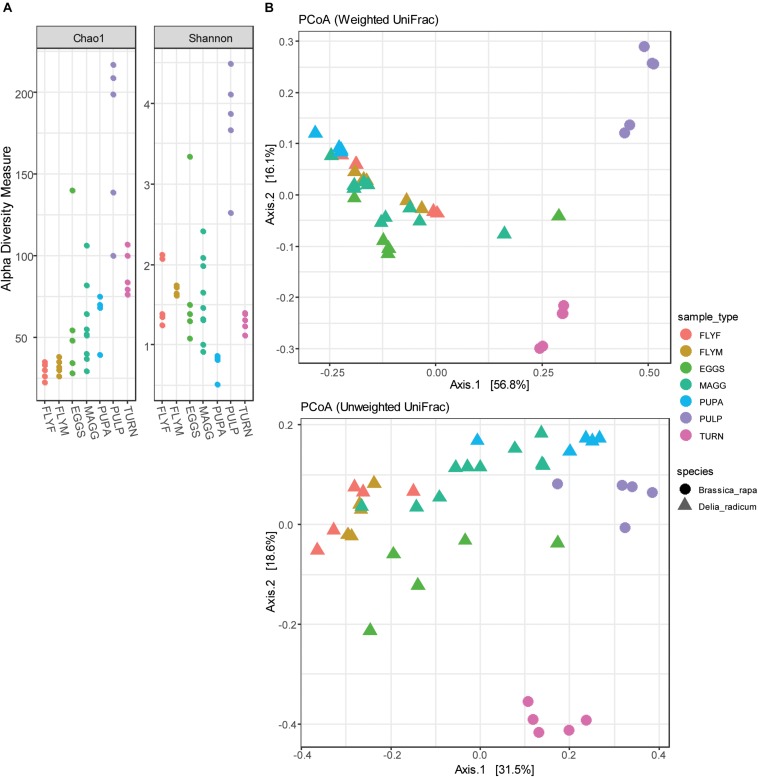
**(A)** Dot plots of the alpha diversity (Chao1 and Shannon-Weaver indices) of *Delia radicum-*associated microbiota and their diet substrates pre feeding (turnip_skin) and post feeding by *D. radicum* larvae (turnip_pulp). **(B)** Principle coordinate analysis of 30 *D. radicum* and 10 *Brassica rapa* samples, based on unweighted and weighted UniFrac distances. MAGG, *D. radicum* larvae; PUPA, *D. radicum* pupae; FLYF, Gravid female adults of *D. radicum*; FLYM, Male adults of *D. radicum*; TURN, Skin of fresh, non-infested *B. rapa* subsp. *rapa*; PULP, Macerated tissue of a *D. radicum*-infested individual of *B. rapa* subsp. *rapa*.

The complexity within samples (alpha diversity) was analyzed by two diversity metrics (Chao1 and Shannon-Weaver) (Figure 5A). Low-abundance reads were included in these analyzes in order to obtain a better indication of sample complexity. The macerated tissue of *D. radicum*–infested turnip showed particularly high complexity by both Chao1 and Shannon-Weaver indices. Bacterial species richness in the microbiome of *D. radicum* is highest in the larval stage, most likely due to the presence of a gut that is filled with decaying plant matter that is, as we show here, characterized by a highly complex bacterial community. The Shannon diversity of pupae was particularly low and can be explained by the high abundance of *Wolbachia* that decreases the evenness of the bacterial community in these samples. The between-sample diversity was visualized by performing principal coordinate analysis (multidimensional scaling), using both weighted and unweighted UniFrac distances (Figure 5B). Assuming a cut-off *p*-value of 0.05, we found significant differences that explained between 25% and 71% of the variation between all sample groups except between the female and male adult fly groups (Table 1). From these results we can conclude that each of the life stages of *D. radicum* has an identifiably unique microbiome. We did not observe significant differences between the microbiome of male and female flies.

**TABLE 1:** Pairwise multilevel comparisons using adonis with 999 permutations.

*P*r(>F)	*D. radicum*	*D. radicum*	*D. radicum*	*D. radicum*	*D. radicum*	Macerated
	Eggs	Larvae	Pupae	Adult females	Adult males	turnip
*D. radicum* Larvae	0.002**					
*D. radicum* Pupae	0.009**	0.001***				
*D. radicum* Adult females	0.010**	0.007**	0.010**			
*D. radicum* Adult males	0.016*	0.001***	0.012*	0.206		
Macerated turnip	0.009**	0.001***	0.012*	0.007**	0.011*	
Fresh turnip	0.008**	0.003**	0.010**	0.010**	0.011*	0.009**

The levels compared are the sample groups that distinguish the *Delia radicum* life stages. *p*-values denoted as (0.010–0.05)*, (0.001–0.010)**, or (0–0.001)***.

## Discussion

In this study we explored the diversity of the microbial community that is associated with the eggs, larvae, pupae and adult life stages of the cabbage root fly *D. radicum*. All developmental life stages contained considerable amounts of *Wolbachia* reads, but also contained a diverse microbial community of other bacteria. Since diet and environment are also factors that influence the microbiome, we included the microbial diversity of the host plant (turnip), and of decayed host plant material that had been infested with *D. radicum* larvae. The decayed plant material was characterized by a very high alpha diversity.

Fresh and macerated turnip samples were furthermore considerably different from the microbial communities of *D. radicum* in structure and composition, but also shared a number of taxa that are possibly transferred between the environment and the host, or vice versa.

*Brassica rapa* subsp. *rapa* is a member of the Brassicaceae family of plants which employ a method of defense against herbivores and pathogenic bacteria by producing the secondary metabolite isothiocyanate (Tierens et al., 2001). Our initial expectation was that the high concentration of isothiocyanates within damaged turnip tissue would only allow isothiocyanate-resistant bacteria to thrive in this environment. Furthermore, many isothiocyanate-resistant strains are within genera that are also shown to have cell wall degrading properties (Basset et al., 2000; Welte et al., 2016b). Instead of a low diversity of strains that are typical cell wall degraders such as *Erwinia*, *Pectobacterium*, or *Pantoea*, these samples contained a flourishing community of Bacteroidetes and Gammaproteobacteria from all different families. Isothiocyanates are volatile and the bioavailability can be expected to differ significantly between the initial moment of *D. radicum* attack and the late-stage infestation marked by complete maceration of the turnip tissue. It is possible that early colonizers are capable of overcoming isothiocyanate-based defenses of the plant, and that a multitude of opportunistic taxa colonize the tissue after the plant defenses have sufficiently diminished over time. The *Serratia* and *Pseudomonas* species that were identified in larval samples could potentially be of great nutritional aid for their hosts. *Serratia plymuthica* 3Rp8, isolated from the rhizosphere of *Brassica napus* L. (Adam et al., 2016), for example, contains two copies of the *saxA* gene (Van den Bosch et al., 2018) that has been implied in overcoming isothiocyanate-based plant defenses in plant pathogenic bacteria (van den Bosch et al., 2019).

All life stages of *D. radicum* were characterized by high abundances of Proteobacterial symbionts, but each was shown to have unique characteristics that could be resolved by comparing various diversity metrics.

Comparisons with other studies that sequenced the 16S rRNA gene of insect-associated gut bacteria show some interesting parallels, as well as dissimilarities.

Bili et al. (2016) previously analyzed the bacterial community of *D. radicum* adults of three different geographic locations in France using 454 pyrosequencing of the V4-V5 region of the bacterial 16S rRNA gene (2016). They reported a relatively low-complexity microbiome dominated by *Wolbachia* or *Wolbachia* and *Gluconacetobacter*, depending on the geographical origin of the flies. The abundance of Firmicutes was low in the fly samples of their study, aligning well with the data presented here. In contrast to the study performed by Bili et al. (2016), we found high abundances of Enterobacteriaceae and we were able to identify multiple Gammaproteobacterial reads which had not been reported previously. *Morganella morganii* is found as a resident of the gut microbiome of the common house fly *Musca domestica* (Gupta et al., 2012) and in the microbiome of healthy bees (Erban et al., 2017) but is also designated as a lethal pathogen in Mexican fruit flies [*Anastrepha ludens* (Loew)] and sand flies [*Lutzomyia longipalpis* (Lutz and Neiva)] (Salas et al., 2017). In our dataset, *Morganella* sp. were abundant in all life stages of *D. radicum*, most of which could not be assigned to a specific species due to the limitation of the sequencing method. In the oriental fruit fly *Bactrocera dorsalis*, it was shown that Proteobacteria dominated immature stages, whereas adult stages were dominated by Firmicutes (Andongma et al., 2015). Although Firmicutes did not quite dominate in *D. radicum* adults, this life stage was the only one where moderate amounts of Firmicute reads were present. In the house fly *M. domestica*, Gammaproteobacteria are dominating larval and adult microbiomes (Gupta et al., 2012; Zhao et al., 2017). Also the microbiomes of other flies harbor a high amount of Gammaproteobacteria, e.g., as found in *Drosophila melanogaster* (Corby-Harris et al., 2007; Cox and Gilmore, 2007). Furthermore, the genus *Comamonas* was reported to be the most abundant in pupae and completely absent in adults of *B. dorsalis* (Andongma et al., 2015). This trend was also observed in the *D. radicum* dataset. 4-6% of non-*Wolbachia* reads in all pupae samples were *Comamonas*, but fly samples contained at most 1%.

For future studies it might be interesting to combine sequencing results from a different laboratory-reared insect species to see whether the variance of microbial diversity is larger between different life stages of one insect, or between two insect species at the same life stage. Studies on house flies have shown that geographical origin and laboratory rearing can have a considerable impact on the microbiome (Park et al., 2019).

The presence of *Wolbachia* has been shown to play a role in determining the microbiome composition in *Drosophila* (Simhadri et al., 2017).

We observed *Wolbachia* reads in all *D. radicum* samples regardless of life stage. In the adult stages, the number of reads assigned to this genus ranged from 25 to 65%, which is markedly lower than the 97% and 80% reported previously (Bili et al., 2016). The vertical transmission rate of *Wolbachia* is 100%, and there is no evidence of reproductive manipulation phenotypes such as feminization, parthenogenesis, male-killing or cytoplasmic incompatibility in *D. radicum* (Lopez et al., 2018). We found particularly high relative abundances of *Wolbachia* in the pupal samples. One possible explanation for this is that larvae shed their gut lining before pupation, evacuating a large part of the gut-associated bacteria (Hammer and Moran, 2019). The distribution of reads over the nine *Wolbachia* ASVs suggests that the presence of multiple genotypes of *Wolbachia* seems unlikely. The artificial OTU richness may stem from technical artifacts such as PCR and/or sequencing errors, or to the limitation of the 16S rRNA gene for taxonomically resolving *Wolbachia* specifically (Ellegaard et al., 2013). The age-dependency of the relative abundance of *Wolbachia* in the gut of the termite *Nasutitermes arborum* was recently described for the first time (Diouf et al., 2018). The relative abundance of *Wolbachia* was shown to be negatively correlated with alpha diversity, suggesting a mutual exclusion from the same environment. Although our sampling methods included homogenization of whole insects, previous studies have indicated that communities from such samples can closely resemble communities that were sampled from the gut alone (Hammer et al., 2014). We therefore suggest that the removal of *Wolbachia* reads *in silico* resulted in a dataset that is a closer representation of the gut lumen of *D. radicum*. For future microbiome analyzes of *Wolbachia-*positive *D. radicum* samples one should consider a more cost-effective approach for reducing the number of *Wolbachia* reads in a sample by specific restriction digestion of the *Wolbachia* 16S rRNA gene, as was recently done in *Drosophila* (Simhadri et al., 2017).

Whether the taxa reported in this study are transient or resident, or even dead or alive, cannot be resolved by the methods presented in this work. Future studies could elucidate the potential of nutritional mutualisms between *D. radicum* and resident microbes.

This work has enabled a deeper understanding of the bacterial players associated with *D. radicum* at different life stages, and of the bacterial nature of the plant rot that is associated with *D. radicum* infestation. As such, it could potentially provide new clues on symbiotic bacteria that could be exploited in biocontrol programs. Elucidation of the transmission patterns and the specific functions of these bacterial players species are interesting platforms for further research.

## Data Availability Statement

The raw data supporting the conclusions of this article are available on NCBI under the BioProject PRJNA573643. Additional data such as the code for generating figures will be made available, without undue reservation, to any qualified researcher.

## Author Contributions

TB and CW designed the research. TB conducted the research, analyzed the data, and wrote the manuscript with input from CW.

## Conflict of Interest

The authors declare that the research was conducted in the absence of any commercial or financial relationships that could be construed as a potential conflict of interest.
