# Supplementary material for: The Microbial Diversity of Cabbage Pest Delia radicum Across Multiple Life Stages
Source: Front Microbiol. 2020 Feb 27;11:315. doi: 10.3389/fmicb.2020.00315 (PMC7056704; doi:10.3389/fmicb.2020.00315)
Supplement: Supplementary file 1 [file Data_Sheet_1.docx]

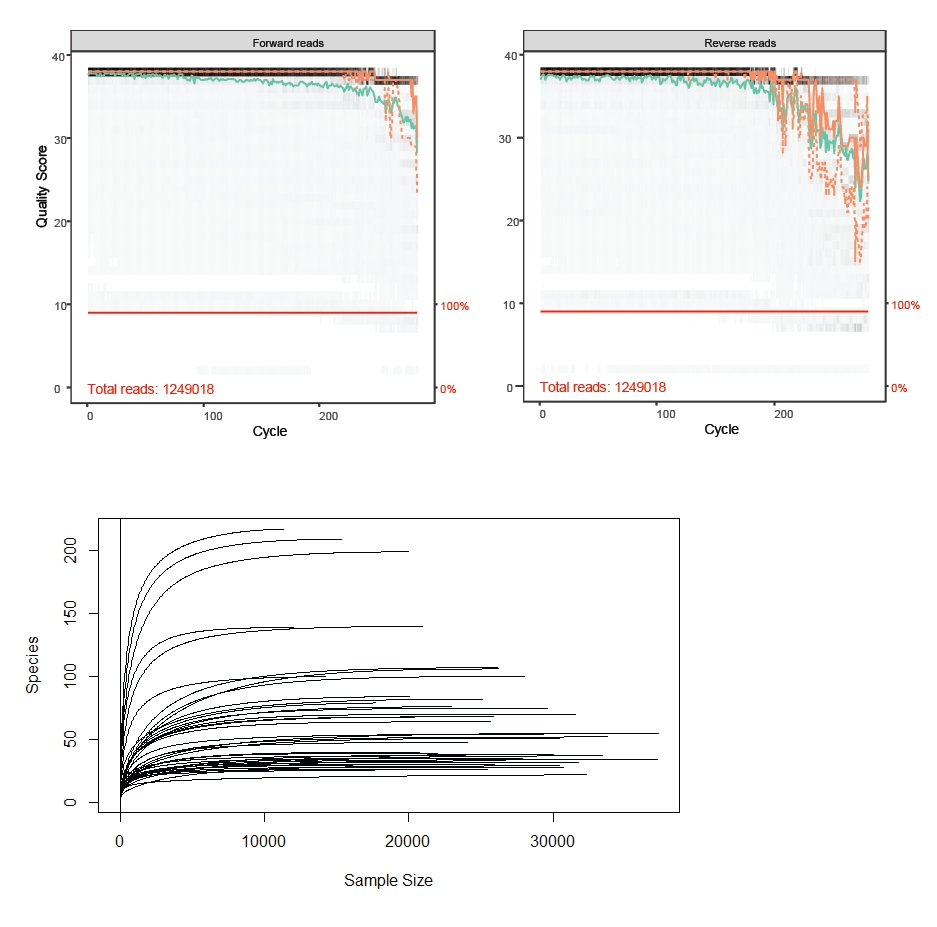


**Supplementary Figure 1**. Quality score (Phred) profiles of all reads, representing aggregated results of 40 samples. Top graph contains only forward reads, bottom graph represents only the paired reverse reads. Green line represent the mean, orange the median, and dashed orange lines represent the 25th and 75th quantiles. Rarefaction curve that shows that all samples have at least 10.000 reads and that all 40 samples have a good coverage of the present microbial diversity.

Supplementary table 1. Metadata table for 40 biological samples for the bacterial community profiling by 16S rRNA gene sequencing.

| #SampleID | NG_Tax_colum_no | sample_ID | extraction_ID | rep_no | species | sample_type | sample_names | date_collection | date_extraction | sample_origin | sample_origin_num | sample_mass | DNA_conc_1 | DNA_conc_2 |
| --- | --- | --- | --- | --- | --- | --- | --- | --- | --- | --- | --- | --- | --- | --- |
| Sample1 | 0 | 11 | 11 | 1 | Delia_radicum | dr_eggs | EGGS1 | 2-15-2018 | 2-16-2018 | eggbox 30-01-2018 (1) | 7 | 0.267 | 11.6 | 11.6 |
| Sample2 | 1 | 12 | 12 | 2 | Delia_radicum | dr_eggs | EGGS2 | 2-15-2018 | 2-16-2018 | eggbox 30-01-2018 (1) | 7 | 0.221 | 4.52 | 24.4 |
| Sample3 | 2 | 13 | 13 | 3 | Delia_radicum | dr_eggs | EGGS3 | 2-15-2018 | 2-16-2018 | eggbox 30-01-2018 (1) | 7 | 0.225 | 16.2 | 14.2 |
| Sample4 | 3 | 14 | 14 | 4 | Delia_radicum | dr_eggs | EGGS4 | 2-15-2018 | 2-16-2018 | eggbox 30-01-2018 (1) | 7 | 0.289 | 3.08 | 4.21 |
| Sample5 | 4 | 15 | 15 | 5 | Delia_radicum | dr_eggs | EGGS5 | 2-15-2018 | 2-16-2018 | eggbox 30-01-2018 (2) | 8 | 0.121 | 5.8 | 5.98 |
| Sample6 | 5 | 31 | 39 | 1 | Delia_radicum | dr_adult_F | FLYF1 | 2-6-2018 | 2-7-2018 | breedbox 1 | 13 | 0.119 | 5.32 | 9.44 |
| Sample7 | 6 | 32 | 40 | 2 | Delia_radicum | dr_adult_F | FLYF2 | 2-6-2018 | 2-7-2018 | breedbox 1 | 13 | 0.122 | 16.5 | 34.4 |
| Sample8 | 4 | 33 | 41 | 3 | Delia_radicum | dr_adult_F | FLYF3 | 2-6-2018 | 2-7-2018 | breedbox 2 | 14 | 0.052 | 4.12 | 8.26 |
| Sample9 | 8 | 34 | 42 | 4 | Delia_radicum | dr_adult_F | FLYF4 | 2-6-2018 | 2-7-2018 | breedbox 2 | 14 | 0.049 | 3.68 | 6.88 |
| Sample10 | 9 | 35 | 44 | 6 | Delia_radicum | dr_adult_F | FLYF5 | 3-20-2018 | 3-20-2018 | breedbox 1 | 13 | 0.119 | 0.98 | 2.18 |
| Sample11 | 10 | 36 | 45 | 1 | Delia_radicum | dr_adult_M | FLYM1 | 2-6-2018 | 2-7-2018 | breedbox 1 | 13 | 0.089 | 8.4 | 16.6 |
| Sample12 | 11 | 37 | 46 | 2 | Delia_radicum | dr_adult_M | FLYM2 | 2-6-2018 | 2-7-2018 | breedbox 1 | 13 | 0.102 | 17.4 | 27.8 |
| Sample13 | 12 | 38 | 47 | 3 | Delia_radicum | dr_adult_M | FLYM3 | 2-6-2018 | 2-7-2018 | breedbox 2 | 14 | 0.06 | 4.2 | 7.08 |
| Sample14 | 13 | 39 | 48 | 4 | Delia_radicum | dr_adult_M | FLYM4 | 2-6-2018 | 2-7-2018 | breedbox 2 | 14 | 0.085 | 3.4 | 7.36 |
| Sample15 | 14 | 40 | 49 | 5 | Delia_radicum | dr_adult_M | FLYM5 | 2-21-2018 | 2-21-2018 | breedbox 2 | 14 | 0.078 | 0.79 | 2.08 |
| Sample16 | 15 | 19 | 26 | 10 | Delia_radicum | dr_larva | MAGG10 | 3-12-2018 | 3-12-2018 | eggbox 30-01-2018 (2) | 8 | 0.072 | 1 | 3.33 |
| Sample17 | 16 | 20 | 27 | 11 | Delia_radicum | dr_larva | MAGG11 | 3-20-2018 | 3-20-2018 | eggbox 30-01-2018 (2) | 8 | 0.126 | 1.69 | 3.36 |
| Sample18 | 17 | 21 | 28 | 12 | Delia_radicum | dr_larva | MAGG12 | 3-20-2018 | 3-20-2018 | eggbox 30-01-2018 (1) | 7 | 0.148 | 1.34 | 3.48 |
| Sample19 | 18 | 22 | 29 | 13 | Delia_radicum | dr_larva | MAGG13 | 3-20-2018 | 3-20-2018 | eggbox 30-01-2018 (1) | 7 | 0.205 | 1.06 | 3.42 |
| Sample20 | 19 | 23 | 30 | 14 | Delia_radicum | dr_larva | MAGG14 | 3-20-2018 | 3-20-2018 | eggbox 05-02-2018 (2) | 9 | 0.149 | 1.06 | 2.32 |
| Sample21 | 20 | 24 | 31 | 15 | Delia_radicum | dr_larva | MAGG15 | 3-20-2018 | 3-20-2018 | eggbox 16-02-2018 (2) | 10 | 0.107 | 1.6 | 3.36 |
| Sample22 | 21 | 25 | 32 | 16 | Delia_radicum | dr_larva | MAGG16 | 3-20-2018 | 3-20-2018 | eggbox 16-02-2018 (2) | 10 | 0.115 | 1.4 | 3.02 |
| Sample23 | 22 | 16 | 21 | 5 | Delia_radicum | dr_larva | MAGG5 | 3-12-2018 | 3-12-2018 | eggbox 30-01-2018 (1) | 7 | 0.146 | 1.48 | 2.9 |
| Sample24 | 23 | 17 | 22 | 6 | Delia_radicum | dr_larva | MAGG6 | 3-12-2018 | 3-12-2018 | eggbox 30-01-2018 (1) | 7 | 0.055 | 1.68 | 3.48 |
| Sample25 | 24 | 18 | 25 | 9 | Delia_radicum | dr_larva | MAGG9 | 3-12-2018 | 3-12-2018 | eggbox 30-01-2018 (2) | 8 | 0.088 | 0.97 | 4.01 |
| Sample26 | 25 | 6 | 6 | 1 | Brassica_rapa | turnip_pulp | PULP1 | 2-5-2018 | 2-5-2018 | eggbox 30-12-2017 | 3 | 0.209 | 2.93 | 7.96 |
| Sample27 | 26 | 7 | 7 | 2 | Brassica_rapa | turnip_pulp | PULP2 | 2-5-2018 | 2-5-2018 | eggbox 30-12-2017 | 3 | 0.276 | 2.3 | 4.76 |
| Sample28 | 27 | 8 | 8 | 3 | Brassica_rapa | turnip_pulp | PULP3 | 2-19-2018 | 2-19-2018 | eggbox 19-01-2018 (2) | 4 | 0.287 | 7.12 | 21.6 |
| Sample29 | 28 | 9 | 9 | 4 | Brassica_rapa | turnip_pulp | PULP4 | 2-19-2018 | 2-19-2018 | eggbox 19-01-2018 (1) | 5 | 0.293 | 7.92 | 23 |
| Sample30 | 29 | 10 | 10 | 5 | Brassica_rapa | turnip_pulp | PULP5 | 2-19-2018 | 2-19-2018 | eggbox 30-01-2018 (2) | 6 | 0.248 | 8.8 | 26.4 |
| Sample31 | 30 | 26 | 34 | 1 | Delia_radicum | dr_pupa | PUPA1 | 2-5-2018 | 2-5-2018 | eggbox 30-12-2017 | 11 | 0.205 | 2.89 | 8.34 |
| Sample32 | 31 | 27 | 35 | 2 | Delia_radicum | dr_pupa | PUPA2 | 2-5-2018 | 2-5-2018 | eggbox 30-12-2017 | 11 | 0.2 | 2.58 | 7.26 |
| Sample33 | 32 | 28 | 36 | 3 | Delia_radicum | dr_pupa | PUPA3 | 2-5-2018 | 2-5-2018 | eggbox 30-12-2017 | 11 | 0.173 | 4.12 | 8.36 |
| Sample34 | 33 | 29 | 37 | 4 | Delia_radicum | dr_pupa | PUPA4 | 2-5-2018 | 2-5-2018 | eggbox 30-12-2017 | 11 | 0.228 | 2.53 | 6.28 |
| Sample35 | 34 | 30 | 38 | 5 | Delia_radicum | dr_pupa | PUPA5 | 2-19-2018 | 2-19-2018 | eggbox 19-01-2018 (2) | 12 | 0.207 | 2.09 | 7.22 |
| Sample36 | 35 | 1 | 1 | 1 | Brassica_rapa | turnip_skin | TURN1 | 1-30-2018 | 1-30-2018 | turnip_1 | 1 | 0.277 | 2.87 | 5.18 |
| Sample37 | 36 | 2 | 2 | 2 | Brassica_rapa | turnip_skin | TURN2 | 1-30-2018 | 1-30-2018 | turnip_1 | 1 | 0.259 | 5.56 | 10.9 |
| Sample38 | 37 | 3 | 3 | 3 | Brassica_rapa | turnip_skin | TURN3 | 1-30-2018 | 1-30-2018 | turnip_2 | 2 | 0.246 | 22.8 | 53.2 |
| Sample39 | 38 | 4 | 4 | 4 | Brassica_rapa | turnip_skin | TURN4 | 1-30-2018 | 1-30-2018 | turnip_2 | 2 | 0.239 | 4.32 | 6.64 |
| Sample40 | 39 | 5 | 5 | 5 | Brassica_rapa | turnip_skin | TURN5 | 1-30-2018 | 1-30-2018 | turnip_2 | 2 | 0.215 | 23.2 | 62 |
